# Polyphenols from *Bacopa procumbens* Nanostructured with Gold Nanoparticles Stimulate Hair Growth Through Apoptosis Modulation in C57BL/6 Mice

**DOI:** 10.3390/pharmaceutics17020222

**Published:** 2025-02-09

**Authors:** Salvador Pérez-Mora, Juan Ocampo-López, María del Consuelo Gómez-García, Sandra Viridiana Salgado-Hernández, Yazmin Montserrat Flores-Martinez, David Guillermo Pérez-Ishiwara

**Affiliations:** 1 Laboratorio de Biomedicina Molecular I, Programas de Doctorado en Ciencias en Biotecnología y Maestría en Biomedicina Molecular, Escuela Nacional de Medicina y Homeopatía (ENMyH), Instituto Politécnico Nacional, Mexico City 07320, Mexico; sperezm1510@alumno.ipn.mx (S.P.-M.); cgomezg@ipn.mx (M.d.C.G.-G.); ssalgadoh1600@alumno.ipn.mx (S.V.S.-H.); yfloresma@ipn.mx (Y.M.F.-M.); 2 Atria Scientific, Departamento de Investigación e Innovación, Av. de los Maestros 452, Nueva Santa María, Azcapotzalco, Mexico City 02800, Mexico; 3 Laboratory of Histology and Histopathology, Academic Area of Veterinary Medicine, ICAp, Universidad Autónoma del Estado de Hidalgo, Tulancingo de Bravo, Pachuca 43600, Mexico; jocampo@uaeh.edu.mx

**Keywords:** BFNB, polyphenols, *Bacopa Procumbens*, hair growth, apoptosis, p53, caspase 3, caspase 9, Bax, Bcl-2, in silico analysis, molecular docking, interactome analysis, pathway enrichment, KEGG, Reactome, compartments

## Abstract

**Background/Objectives**: Alopecia is a hair disorder with a significant impact on quality of life, and its incidence has been increasing in recent years. Current therapeutic options are limited and may cause adverse side effects, highlighting the need to develop safer and more effective formulations. Therefore, the objective of this study was to evaluate the effect of a formulation based on the bioactive fraction of *Bacopa procumbens* (BFNB), conjugated with gold nanoparticles, on hair growth through the modulation of apoptosis in C57BL/6 mice. **Methods**: The potential biological activities of the secondary metabolites of *B. procumbens* present in BFNB were analyzed in silico. In vivo experiments evaluated the expression of pro-apoptotic markers p53, caspase 3-p11, caspase 9-p10, and Bax, as well as anti-apoptotic marker Bcl-2, through Western blotting. Immunohistochemistry further assessed the expression and localization of some of these markers. Additionally, molecular docking and interactomic analyses were performed, complemented by functional enrichment, to explore molecular pathways modulated by the evaluated proteins. **Results**: In silico analyses suggested that BFNB metabolites are involved in the modulation of hair growth, hair fragility, and apoptosis. This finding was supported by in vivo experiments in mice, where BFNB significantly decreased the expression of p53, caspase 3-p11, caspase 9-p10, and Bax while increasing Bcl-2 levels. Immunohistochemistry showcased a reduction in pro-apoptotic markers in dermal and follicular bulb cells. Furthermore, molecular docking studies identified BFNB metabolites as potential direct modulators of these key proteins, strengthening evidence of their role in apoptotic regulation. The interactomic analysis highlighted 50 proteins associated with apoptosis, and functional enrichment underscored key processes such as p53 signaling, regulation of the apoptosome, and mitochondrial membrane involvement in the intrinsic apoptosis mechanism, among other pathways. **Conclusions**: This study demonstrates that BFNB effectively modulates apoptosis through key molecular mechanisms, highlighting its potential as an innovative therapy for promoting hair growth.

## 1. Introduction

Hair, beyond its aesthetic role, serves essential functions, such as scalp protection and thermal regulation. Additionally, it contributes to defense against external elements, the production of pheromones, and the secretion of apocrine sweat and sebum, in addition to playing a crucial role in social interactions [1]. Alopecia, defined as abnormal or pathological hair loss, affects a significant portion of the world’s population, including both men and women, and has garnered increasing interest in the fields of medical and aesthetic research due to its profound impact on people’s quality of life, affecting their self-esteem and emotional well-being [2].

Statistically, it is estimated that approximately 50% of men and 30% of women experience some degree of hair loss during their lifetime, with androgenic alopecia being the most frequent form [3]. According to data from the American Academy of Dermatology [4], this condition is the leading cause of hair loss, while alopecia areata, which affects approximately 2% of people worldwide, ranks as the second most common form.

The hair cycle is composed of three primary phases: anagen (growth), catagen (regression), and telogen (rest) phases [5]. In humans, the anagen phase lasts 2 to 6 years and is marked by active hair growth, followed by the catagen phase, a brief 2- to 3-week transitional phase characterized by cell apoptosis. Lastly, the telogen phase spans 2 to 4 months, representing a resting period before hair shedding and the cycle’s renewal [6].

The phases of the hair cycle can be disrupted by various factors, including genetic, hormonal, and nutritional imbalances; UV radiation exposure; aging; autoimmune reactions; stress; and the side effects of medications such as those used in cancer therapies. These disruptions lead to alopecia, which is characterized by progressive hair thinning and, ultimately, shedding [6,7]. Consequently, the development of new treatments that are both more effective and have fewer adverse effects remains a priority.

Advancements in alopecia treatment focus on innovative strategies addressing genetic and environmental factors influencing the hair cycle. Among these, vitamins (A, B7, C, D, and E) have emerged as key modulators of hair growth, stimulating pathways such as the WNT/β-catenin and VEGF signaling pathways, regulating the cell cycle, strengthening keratin, promoting collagen synthesis, and protecting follicles from oxidative damage [8]. In parallel, CRISPR/Cas9 technology enables precise gene editing of targets like *SRD5A2*, *FGF5*, and *VDR*, modulating key pathways such as the WNT/β-catenin, VEGF, BMP2/4, and *PI3K*/*AKT* pathways. Experimental models show its potential to prolong the anagen phase, enhance follicle density, and correct abnormalities, offering innovative and personalized therapeutic options [9].

Current pharmacological treatments include topical agents such as minoxidil and oral therapies like 5α-reductase inhibitors (primarily finasteride) and baricitinib, which was recently approved by the FDA for alopecia areata. However, these options have limitations regarding long-term efficacy and adverse side effects [10,11,12]. From this perspective, the development of therapies based on natural compounds, such as those derived from medicinal plants, represents a promising strategy. These compounds contain bioactive molecules capable of promoting the anagen phase and reducing follicular apoptosis, offering a more natural and potentially less invasive approach to addressing alopecia [13,14].

Within this framework, apoptosis plays a crucial role in regulating the hair cycle, particularly during the catagen and telogen phases of hair follicles. Under normal conditions, this process ensures hair renewal and maintains the balance of follicle numbers in the scalp. However, an altered apoptosis process in the follicular cells or a shortened anagen phase can disrupt growth, reduce density, and impair hair functionality, contributing to the development of alopecia [15]. Therefore, modulating the apoptotic balance emerges as a key therapeutic strategy to combat hair loss and promote regeneration [16].

Indeed, Wang et al. [16] emphasize that modulating apoptosis revitalizes the hair cycle, improves hair quality, and extends its growth phase. This includes interventions aimed at reducing pro-apoptotic markers such as p53, caspase 3 (Casp3), and caspase 9 (Casp9) while promoting the expression of anti-apoptotic markers such as Bcl-2.

Plant extracts such as *Alnus sibirica* [17] and ginseng (*Panax ginseng*) [18] have demonstrated significant potential in stimulating hair growth and counteracting the regressive phases of the hair cycle through the modulation of the apoptosis–cell survival axis. Similarly, various plant-derived compounds, including quercitrin [19], caffeine [20], epigallocatechin gallate [21], procyanidin B2 [22], procyanidin B-3 [23], decursin [24], oleuropein [25], and ginsenoside Rb1 [26], among others, have been reported to modulate hair growth by regulating follicular phases. In this context, *Bacopa procumbens* (*B. procumbens*), the plant used in our study, emerges as a promising alternative due to its secondary metabolites with bioactive properties.

Recently, in an initial study related to hair growth, we also demonstrated that the aqueous–ethanolic extract of *B. procumbens*, nanostructured and conjugated with gold nanoparticles, incorporated into a dermatological-grade lipophilic formulation (referred to as BFNB) enhanced hair growth in C57BL/6 mice. This effect was achieved through the modulation of growth factors such as epidermal growth factor (EGF) and fibroblast growth factor 7 (FGF7), as well as the activation of the PI3K/AKT/β-catenin signaling pathway. Additionally, cell cycle activation was observed, as evidenced by the expression of proteins such as PCNA, KI-67, Cyclin D1, and Cyclin E [27].

Building on these findings, in the present study (the second phase of our research), we evaluated the effect of the formulation on apoptosis modulation. The results are promising, offering a new perspective on the use of nanostructured natural compounds to regulate apoptosis and promote hair growth. This could lay the foundation for innovative alopecia by promoting hair regrowth.

## 2. Materials and Methods

### 2.1. Prediction of BFNB Metabolite Activities in Processes Related to Hair Growth and the Modulation of Apoptosis

Based on the metabolites identified in the bioactive fraction of *B. procumbens* previously reported by our research team [28], we selected only those with abundance values exceeding 1%. Under this criterion, our analysis included a total of sixteen secondary metabolites (Supplementary Figure S1). The SMILES codes for each metabolite were retrieved utilizing the PubChem platform (https://pubchem.ncbi.nlm.nih.gov/, accessed on 3 July 2024) (Table 1).

Subsequently, to predict their potential molecular targets or biological activities, the SMILES codes of each BFNB metabolite were entered into the PASS Online server, version 2.0, available in Way2Drug (https://www.way2drug.com/passonline/, accessed on 4 July 2024). Predictions were performed with default parameters, focusing on activities with scores >0.5, which indicate a reasonable probability of biological activity based on the model’s training data. The PASS Online server predicts biological activities using statistical models based on machine learning. These predictions are based on the structural similarity of the analyzed compounds to those in its database, which have documented biological activities. Using this information, it calculates probabilities for various categories or potential activities associated with the metabolites [29]. Activities related to hair growth and apoptosis, involving key proteins like p53 and caspases, were included. Results were visualized as a heat map in Microsoft Excel 365 (Microsoft Corporation, Redmond, WA, USA) using a blue gradient scale for clarity.

### 2.2. Preparation of the BFNB Formulation

The bioactive fraction of *B. procumbens* corresponds to an aqueous–ethanolic extract, which was nanostructured and conjugated with gold nanoparticles following the protocol described by Martínez-Cuazitl et al. [30]. Characterization of the synthesis of gold nanoparticles, as well as their conjugation with the plant extract and stability, was carried out by the same authors using UV-Vis, ATR-FTIR, DLS, zeta potential, and TEM analysis.

BFNB was obtained following the protocol described by Pérez et al. [27], which provides a detailed explanation of the process. Briefly, gold nanoparticles were synthesized using the Turkevitch method [31] and functionalized with the bioactive fraction of *B. procumbens* at a concentration of 0.8 mg/mL. This mixture was subsequently incorporated into a lipophilic dermatological serum, a standard generic base containing glycerin, caprylic triglycerides, and other excipients, resulting in the final product [27].

### 2.3. Experimental Animals

Male C57BL/6 mice were used and maintained under controlled conditions to ensure their well-being. Environmental conditions included a temperature of 22 ± 1 °C, relative humidity of 55 ± 5%, and 12 h light/dark cycles. The animals had ad libitum access to drinking water and a standard balanced diet. After shaving the dorsal and head areas, the treatments, including the vehicle (a formulation without *B. procumbens* extract conjugated with gold nanoparticles), 2% minoxidil, and the BFNB formulation, were applied topically to the mice for 30 days, with each application performed every 24 h. The experimental protocols were approved by the Bioethics Committee of ENMH-IPN under registration number CBE/005/2021 and were conducted in accordance with the provisions of Mexican Official Standard NOM-062-ZOO-1999, ensuring compliance with regulations for the care and use of animals in research [32].

### 2.4. Western Blotting

In C57BL/6 mice, dorsal and head skin samples were collected fifteen days after treatment, a time point identified in a previous study [27] as critical for observing significant hair growth, including increased thickness, length, and follicle count. Earlier time points (day ten) showed only pigmentation, while follicular involution began after day eighteen, making day fifteen ideal for evaluating molecular pathways involved in hair growth.

Once the skin samples were obtained, they were homogenized using an Ultra-Turrax T18 (IKA, San Diego, CA, USA) at 9000 rpm for 5 min in RIPA buffer supplemented with protease inhibitors (Complete™, Merck, Darmstadt, HE, Germany). The homogenates were centrifuged at 13,000 rpm for 15 min at 4 °C, and the soluble fraction was collected. Protein quantification was performed employing the Bradford method [33].

For Western blot analysis, 20 µg of protein per group was separated on a 12% SDS-PAGE gel. Proteins were transferred to PVDF membranes (Merck, Darmstadt, HE, Germany), which were blocked with 2% BSA for 1 h at room temperature. The membranes were incubated with primary antibodies (1:15,000 dilution) and secondary antibodies (1:30,000 dilution), each for 30 min at room temperature. Detection was performed utilizing chemiluminescence with an Immobilon Western Chemiluminescent HRP Substrate kit (Sigma, Burlington, MA, USA).

The primary antibodies used in the study included anti-p53 (#sc-47698), anti-caspase 3-p11 (#sc-271759), anti-caspase 9-p10 (#sc-7885), anti-Bcl-2 (#sc-130308), and anti-Bax (#sc-7480). Anti-GAPDH (#sc-32233) was used as an endogenous control. All primary antibodies were purchased from Santa Cruz Biotechnology (Santa Cruz Biotechnology, Inc., Dallas, TX, USA). This company specifically indicates that Casp3-p11 and Casp9-p10 are generated through proteolysis of the inactive precursors and represent markers of the mature and, consequently, active forms of these caspases.

The secondary antibodies, conjugated with horseradish peroxidase (HRP), were anti-mouse IgG (#115-035-062) and anti-rabbit IgG (#111-035-003), selected according to the origin of the corresponding primary antibody. Both secondary antibodies were obtained from Jackson ImmunoResearch (West Grove, PA, USA).

### 2.5. Immunohistochemistry

Longitudinal and transverse histological sections (8 µm thick) were obtained from the dorsal skin of mice treated for 15 days and processed for immunohistochemical assays as described by Pérez et al. [27], with slight modifications. Samples were deparaffinized, rehydrated through a descending ethanol series, and subjected to epitope retrieval with citrate solution (Sigma-Aldrich, St. Louis, MO, USA) using a pressure cooker (Oster, FlavorMaster™, Owosso, MI, USA) for 10 min. Endogenous peroxidase activity was blocked with peroxidase blocking buffer from a Bio SB kit (Santa Barbara, CA, USA), and nonspecific binding was reduced with 5% BSA (Sigma-Aldrich) for 30 min.

Sections were incubated with primary antibodies (anti-p53, anti-caspase 3-p11, and anti-caspase 9-p10; 1:200) for 1 h, followed by a secondary antibody from a Mouse/Rabbit PolyDetector DAB HRP Brown kit. Visualization was performed using DAB substrate, and nuclei were counterstained with Mayer’s hematoxylin (Sigma-Aldrich, St. Louis, MO, USA). Samples were dehydrated, cleared in xylene, and mounted with GVA-mount reagent (Zymed, San Francisco, CA, USA). Images were captured with a Nikon microscope (10x and 40x objectives), and relative protein expression was quantified using ImageJ software, version 1.54 (NIH, Bethesda, MD, USA).

### 2.6. Three-Dimensional Modeling and Structural Validation of Apoptosis-Associated Proteins

Due to the lack of complete crystallized structures for the p53, Casp3, and Casp9 proteins, it was necessary to generate high-quality 3D models to ensure the validity of the results. The 3D structures of p53, Casp3, and Casp9 were constructed using SWISS-MODEL, version 2024 (https://swissmodel.expasy.org/, accessed on 7 July 2024). Amino acid sequences were retrieved from UniProt (https://www.uniprot.org/, accessed on 7 July 2024) using the identifiers P02340 (p53), P02340 (Casp3), and Q8C3Q9 (Casp9), all corresponding to *Mus musculus* (*M. musculus*). These sequences were uploaded to the server to generate monomeric structures in .pdb format.

To obtain the 3D structures of the analyzed proteins, templates from the Protein Data Bank (PDB) (https://www.rcsb.org/, accessed on 7 July 2024) were employed. For p53, the crystallographic structure of the p53/RNA polymerase II complex from *Homo sapiens* (*H. sapiens*) (ID: 6XRE) was used, as it shares 100% sequence identity with the *M. musculus* protein according to SWISS-MODEL. For Casp3, the crystallographic structure from *H. sapiens* (ID: 5I9B) was selected, showing 86.28% sequence identity between the two species. Finally, for Casp9, the cleaved structure lacking the CARD domain from *H. sapiens* (ID: 1JXQ) was chosen, with a 79.49% sequence similarity to the *M. musculus* Casp9.

The generated models were subsequently subjected to rigorous validation employing bioinformatics tools, following methodologies previously reported by Pérez et al. [34], Dorantes et al. [35], and Soto-Sánchez et al. [36].

Structural quality analysis was conducted using the PDBsum server, version 2024 (https://www.ebi.ac.uk/thornton-srv/databases/pdbsum/Generate.html, accessed on 10 July 2024), to evaluate φ and ψ angles via Ramachandran plots and assess stereochemical conformation. ProSA-web, version 2007 (https://prosa.services.came.sbg.ac.at/prosa.php, accessed on 11 July 2024), was used to compare Z-scores with crystallographic structures, assessing overall quality and energy profiles. ERRAT, version 6 (https://saves.mbi.ucla.edu/, accessed on 11 July 2024), analyzed atomic interactions within the 3D models, providing a quality factor for model reliability. These tools ensured the accuracy of the generated models.

### 2.7. Prediction of Pockets

We predicted the binding pockets of the p53, Casp3, and Casp9 proteins, as these sites were considered a key criterion for selecting ligands capable of effectively interacting with them. These predictions were performed with the PrankWeb: Ligand Binding Site Prediction server based on P2RANK (https://prankweb.cz/, accessed on 15 July 2024). The previously generated 3D models of p53, Casp3, and Casp9 in .pdb format were uploaded to the server. The analysis was conducted by applying the default configuration with the “Default model with conservation” option, which considers both the protein geometry and the evolutionary conservation of residues [37].

For each protein, the pocket with the highest probability score was selected, ensuring a high level of confidence in the prediction. This approach enabled the identification of potential binding sites with high precision, supporting the reliability of the model for subsequent protein–ligand interaction analyses.

### 2.8. Molecular Docking Simulations

To predict which BFNB metabolites could bind and modulate p53, Casp3, and Casp9 proteins, molecular docking studies were performed. For this purpose, the PyRx—Python Prescription Virtual Screening Tool, version 0.8 (Center for Computational Structural Biology, CCSB, La Jolla, CA, USA), was employed.

Ligands were prepared with Open Babel (integrated within the same program), which involved minimizing their conformational energy, assigning partial charges (Gasteiger), and identifying rotatable bonds to define their flexibility. The ligands were then converted to .pdbqt format, ensuring they were optimized for docking by incorporating these features, along with their 3D coordinates.

Similarly, protein preparation included conversion to .pdbqt format, removal of non-essential molecules such as water, addition of polar hydrogen, and assignment of partial charges (Kollman). Proteins were treated as rigid structures during the simulations to model their interactions with the ligands accurately.

Once the sixteen BFNB metabolites and the p53, Casp3, and Casp9 proteins were prepared, blind molecular docking simulations were carried out employing AutoDock Vina under the software’s recommended default conditions.

For blind docking, specific grid boxes were generated for each protein. For p53, the grid center coordinates were X: 217.146, Y: 146.933, and Z: 221.8716, with dimensions of X: 101.6595, Y: 57.4411, and Z: 110.9642. For Casp3, the center was X: −40.717, Y: −12.107, and Z: −2.9391, with dimensions of X: 56.3176, Y: 49.5201, and Z: 46.2729. For Casp9, the center coordinates were X: 35.2390, Y: 14.9358, and Z: 99.6682, with dimensions of X: 70.3048, Y: 43.5944, and Z: 50.6581. These configurations, performed in blind mode, allowed the ligands to bind freely to any potential site on the entire protein, rather than being restricted to a specific site or the predicted pocket, thereby increasing the probability of identifying relevant interactions.

After completing the molecular docking simulations, Excel files were generated containing binding affinity values in kcal/mol and docking models in .pdb format.

Selection criteria were based on two main aspects: (1) the most favorable conformer of each BFNB metabolite (that is, the one with the most negative binding affinity) was selected, and (2) it was ensured that the selected conformer was located within the predicted pocket of each protein. To validate the in silico results, reference inhibitors documented in the literature, as shown in Table 2, were used to compare the docking outcomes.

### 2.9. Intermolecular Analysis

The intermolecular analysis was performed employing Discovery Studio Visualizer, version 24.1 (Dassault Systèmes BIOVIA, San Diego, CA, USA), generating 2D diagrams that illustrate the interactions and types of bonds present in the complexes. Additionally, the Protein-Ligand Interaction Profiler (PLIP) server (https://plip-tool.biotec.tu-dresden.de/plip-web/plip/index, accessed on 18 September 2024, Dresden, Germany) was used to complement and enrich the information on intermolecular interactions.

### 2.10. Visualization of 3D Models and Molecular Interactions

PyMOL, version 2.5.0 (Schrödinger, LLC, New York, NY, USA), was used to generate images of the 3D models, molecular docking results, and ligand interactions with key amino acids involved in binding.

### 2.11. Interactome Analysis and Enrichment of Apoptosis-Related Molecular Pathways

To identify potential functional and regulatory interactions among the proteins studied in vivo via Western blot (p53, Casp3, Casp9, Bax, and Bcl-2), we constructed an interactome utilizing STRING, version 12 (https://string-db.org/, accessed on 27 November 2024), for the *M. musculus* species.

The network was expanded by comparing these proteins with the *M. musculus* proteome, applying an interaction probability threshold ≥ 70% and limiting the network to the 50 proteins with the highest interaction potential.

To supplement the interactome, an enrichment analysis was conducted utilizing three platforms integrated into STRING: KEGG pathways enrichment, Reactome pathways enrichment, and subcellular localization (COMPARTMENTS) enrichment. These tools, connected to databases and libraries, enable the exploration of protein–protein interactions, the identification of relevant molecular pathways (KEGG and Reactome), and the determination of the subcellular localization of proteins (COMPARTMENTS).

Similarly (and to complement these analyses), we performed a second interactomic and functional enrichment analysis with the GeneMANIA server, version 2024 (https://genemania.org/, accessed on 29 November 2024). This analysis is based on gene-level associations, in contrast to STRING, which conducts analyses at the protein level. Additionally, we evaluated the same association among the proteins of interest and expanded the network using an interaction probability threshold ≥ 70% within the *M. musculus* proteome.

### 2.12. Graphical and Statistical Analysis

Graphs were generated with GraphPad Prism, version 8.0.1 (GraphPad Software, San Diego, CA, USA). Statistical analysis was performed utilizing one-way and two-way ANOVA, followed by Tukey’s multiple comparisons test. Significance levels are reported in the APA (American Psychological Association) format: * *p* ≤ 0.033, ** *p* ≤ 0.002, *** *p* ≤ 0.001, and # *p* ≤ 0.033. Results are expressed as mean ± standard deviation (SD) based on at least three independent experiments.

## 3. Results

### 3.1. BFNB Metabolites Exhibit Potential In Silico Activity in the Modulation of Hair Growth and Fragility, as Well as Apoptotic Pathways

The analysis identified three main categories where BFNB metabolites may exhibit activity of interest: hair treatment, hair fragility, and modulation of apoptotic pathways. In the hair treatment category, six metabolites were identified as potential candidates for alopecia treatment. Regarding hair fragility, five metabolites were identified as having potential biological activity. Finally, in the apoptotic pathway category, eight metabolites were highlighted for their association with this critical biological process.

**Figure 1:**
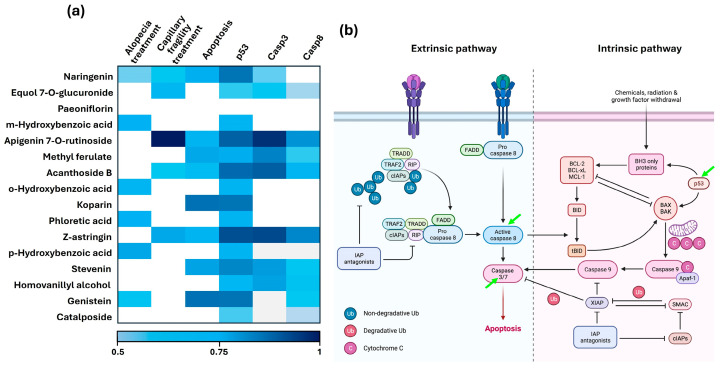
In silico prediction of the potential activities of BFNB metabolites in the treatment of hair growth and fragility, as well as their associations with protein targets involved in apoptosis. (**a**) Heatmap showing the probabilities obtained from the PASS Online server (values ≥ 0.5). The intensity of the blue color indicates the probability of modulatory activity, while white squares represent probabilities ≤ 0.5 or the absence of activity. (**b**) Schematic representation of the molecular pathways of apoptosis obtained from BioRender (https://www.biorender.com/, accessed on 3 July 2024). Green arrows indicate the target proteins of BFNB metabolites predicted to be involved in the apoptotic pathways, including both the extrinsic and intrinsic pathways.

Specifically, when evaluating targets related to apoptotic pathways, fifteen metabolites were identified as potential regulators of the p53 protein. Additionally, eight and nine metabolites were associated with the activity of Casp3 and Casp8, respectively, reinforcing their potential in the modulation of key proteins in the intrinsic and extrinsic apoptotic pathways (Figure 1a).

Notably, BFNB metabolites were observed to potentially modulate two or three key molecular targets associated with intrinsic and extrinsic apoptotic pathways: p53, Casp3, and Casp8. Among these metabolites, naringenin stood out for its potential use in the treatment of alopecia and hair fragility, as predicted by the server, and for its ability to influence apoptosis by specifically targeting p53 and Casp3. Interestingly, metabolites such as equol-7-O-glucuronide, apigenin-7-O-rutinoside, acanthoside B, and Z-astringin demonstrated potential not only for treating hair fragility but also for simultaneously acting on p53, Casp3, and Casp8. Additionally, these three protein targets may also be modulated by methyl ferulate, stevenine, and homovanillic alcohol. In contrast, paeoniflorin did not show potential activity on the evaluated targets, likely due to the absence of corresponding records in the database.

The combined action of these metabolites highlights their potential as multifaceted modulators and suggests a possible synergistic effect by targeting multiple key apoptotic pathways. These findings indicate that BFNB metabolites could influence both intrinsic and extrinsic apoptotic pathways in a complementary manner (Figure 1b).

### 3.2. BFNB Downregulates the Expression of Proteins Involved in Apoptosis

Upon evaluating p53 protein expression, the antibody used in the study detected two bands corresponding to the 50 kDa and 53 kDa isoforms. In mice treated with the BFNB formulation, both isoforms showed a significant reduction in expression compared to the control group, with decreases of 0.46- and 0.50-fold in the dorsal region and 0.42- and 0.40-fold in the head, respectively.

Similarly, Casp3-p11 levels significantly decreased with BFNB treatment, showing reductions of 0.45-fold in the dorsal region and 0.51-fold in the head compared to the control group. Casp9-p10 levels also showed significant reductions, decreasing by 0.63-fold in the dorsal region and 0.44-fold in the head.

**Figure 2:**
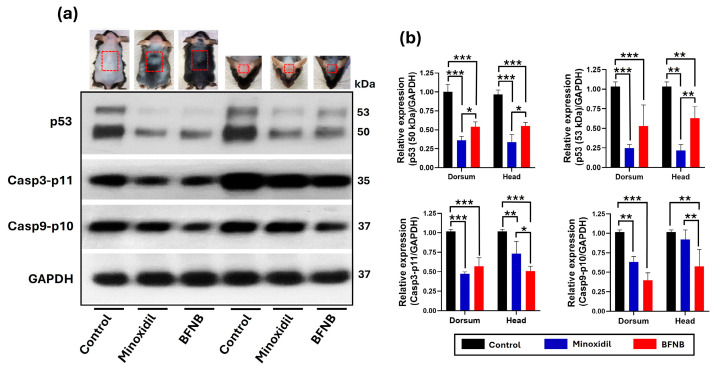
Effect of BFNB on the expression of key proteins involved in the intrinsic apoptosis pathway. (**a**) Western blot analysis of p53, Casp3-p11, and Casp9-p10, with GAPDH used as the endogenous control. The mouse images correspond to results from our previous study [27]. The red box indicates the specific area from which the samples were collected for protein expression analysis. (**b**) Relative quantification of protein expression normalized to GAPDH. Statistical analysis was performed using two-way ANOVA followed by Tukey’s test based on the mean protein expression from three mice per group. Significant differences are indicated as (*), * *p* ≤ 0.033, ** *p* ≤ 0.002, and *** *p* ≤ 0.001.

It is noteworthy that, while minoxidil was more effective in reducing p53 expression in both regions (dorsal and head) compared to BFNB, the BFNB formulation was more efficient than minoxidil in reducing Casp3-p11 and Casp9-p10 levels in both analyzed regions (Figure 2 and Table 3).

### 3.3. BFNB Enhances Bcl-2 Protein Expression and Reduces Bax Levels

A consistent effect of both BFNB and minoxidil on the apoptosis process was observed. Both treatments increased Bcl-2 protein expression and decreased Bax levels compared to the control group. Specifically, BFNB increased Bcl-2 expression by 4.23-fold in the dorsal region and 2.77-fold in the head compared to the control, while Bax levels were reduced by 0.28-fold and 0.39-fold in the same regions.

Additionally, BFNB demonstrated a greater effect in enhancing Bcl-2 expression, with increases of 2.43-fold in the dorsal region and 1.20-fold in the head compared to minoxidil. Regarding Bax, BFNB showed a significant increase only in the head compared to minoxidil.

**Figure 3:**
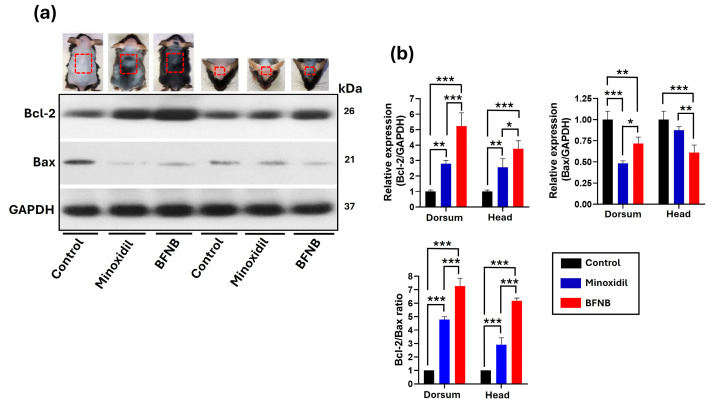
Modulation of the pro- and anti-apoptotic balance. (**a**) Western blot analysis of Bcl-2 and Bax, with GAPDH used as the endogenous control. The mouse images correspond to results from our previous study [27]. The red box indicates the specific area from which the samples were collected for protein expression analysis. (**b**) Relative quantification of Bcl-2 and Bax expression normalized to GAPDH, as well as the Bcl-2/Bax ratio. Statistical analysis was performed using two-way ANOVA followed by Tukey’s test based on the mean protein expression from three mice per group. Significant differences are indicated as (*), * *p* ≤ 0.033, ** *p* ≤ 0.002, and *** *p* ≤ 0.001.

These findings align with the Bcl-2/Bax ratio; in this survival–apoptosis axis, anti-apoptotic protein Bcl-2 was prominently expressed in mice treated with BFNB compared to the other two experimental groups. This effect is reflected in a Bcl-2/Bax ratio 6.28-fold higher in the dorsal region and 5.18-fold higher in the head compared to the control group. The ratio also showed significant differences relative to the minoxidil-treated group, consistently favoring BFNB in both evaluated regions (Figure 3 and Table 4).

### 3.4. BFNB Downregulates p53 and Caspase Activation in Hair Follicles and Dermal Cells

Due to the consistent hair-growth effect of BFNB and the greater tissue availability in the dorsal region for immunohistochemical analysis, we focused exclusively on this area.

In longitudinal sections, p53, Casp3-p11, and Casp9-p10 proteins were localized in both dermal cells and the periphery of the hair follicle in control mice. In contrast, the expression levels of these proteins were significantly reduced in mice treated with minoxidil or BFNB. Specifically, BFNB promoted reductions of 0.68-, 0.61-, and 0.66-fold in the levels of p53, Casp3-p11, and Casp9-p10, respectively, compared to the control group. Similarly, minoxidil significantly reduced the levels of p53 and Caspa3-p11 compared to the control group.

**Figure 4:**
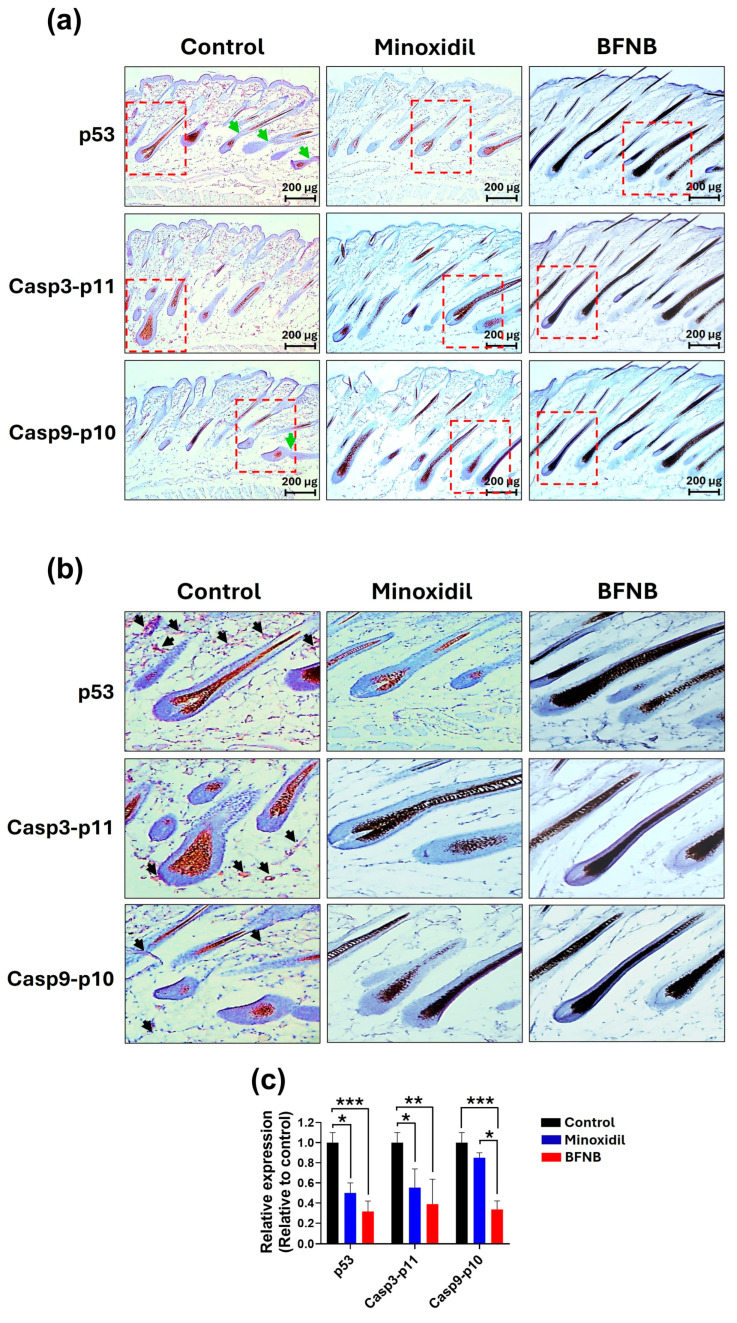
Immunodetection of proteins associated with the intrinsic apoptosis pathway in dorsal longitudinal sections of mice. (**a**) Immunohistochemical assays performed on samples collected fifteen days post treatment. Green arrows indicate marked discontinuity in imbricated cells, a typical process of the catagen phase. (**b**) Close-ups of the areas highlighted in the red boxes shown in (**a**). Black arrows point to the deposition of the enzymatic reaction product resulting from the immunohistochemical assay. (**c**) Quantification of relative expression, calculated as the mean pixel values from three independent samples per experimental group, normalized to the control group. Statistical analysis was performed using two-way ANOVA followed by Tukey’s test. Significant differences are indicated as (*), * *p* ≤ 0.033, ** *p* ≤ 0.002, and *** *p* ≤ 0.001.

The only significant difference observed between BFNB and minoxidil was in the levels of Casp9-p10, which were lower in mice treated with BFNB (Figure 4 and Table 5).

**Figure 5:**
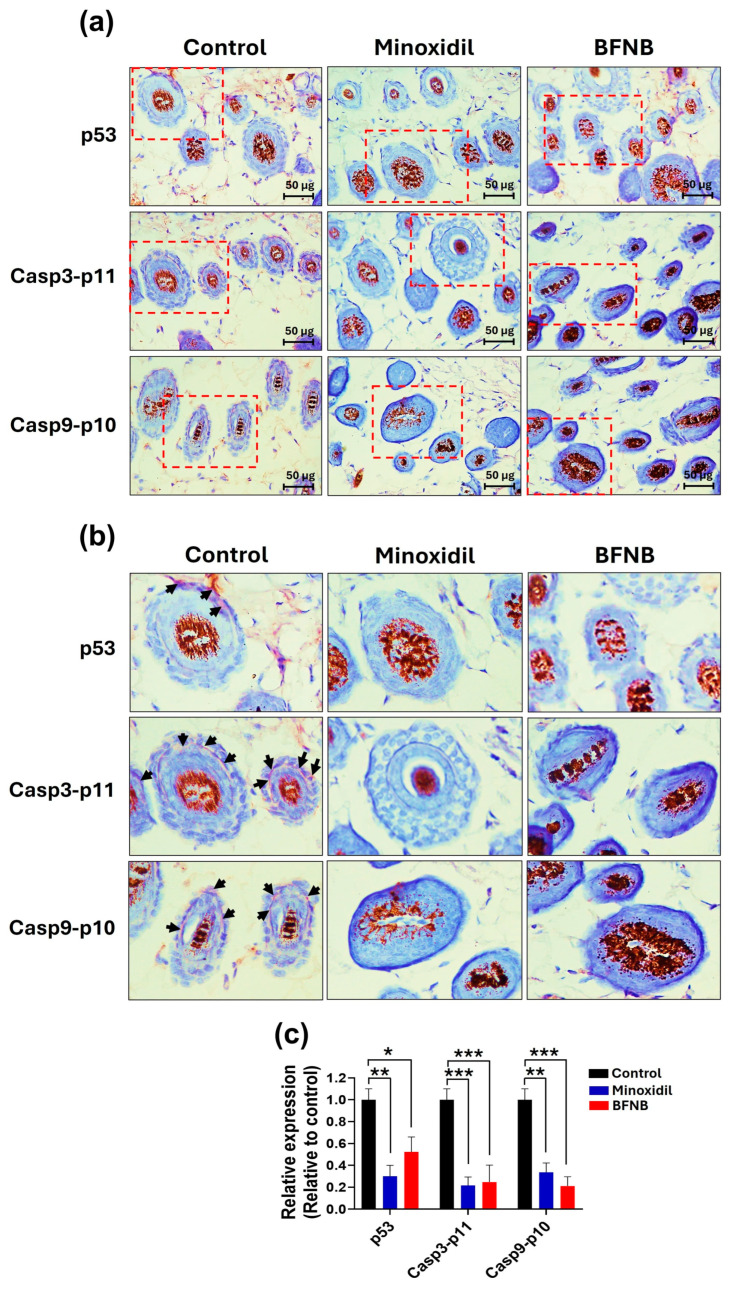
Immunodetection of p53, Casp3-p11, and Casp9-p10 proteins in transverse dorsal sections of mice. (**a**) Immunohistochemical assays performed on samples collected fifteen days post treatment. (**b**) Close-ups of the areas highlighted in the red boxes shown in (**a**). Black arrows indicate the deposition of the enzymatic reaction product resulting from the immunohistochemical assay. (**c**) Quantification of relative expression, calculated as the mean pixel values from three independent samples per experimental group, normalized to the control group. Statistical analysis was performed using two-way ANOVA followed by Tukey’s test. Significant differences are indicated as (*), * *p* ≤ 0.033, ** *p* ≤ 0.002, and *** *p* ≤ 0.001.

Consistent with this result, transverse sections showed that p53, Casp3-p11, and Casp9-p10 were predominantly expressed in the peripheral regions of hair follicles in the control group. In contrast, mice treated with BFNB exhibited a significant reduction in the expression of these proteins, with decreases of −0.48-, −0.75-, and −0.79-fold, respectively, compared to the control group. A similar effect was observed in mice treated with minoxidil, highlighting the ability of both treatments to downregulate pro-apoptotic markers in these regions (Figure 5 and Table 6).

### 3.5. Three-Dimensional Modeling and Structural Validation of p53, Casp3, and Casp9

The 3D models were generated using the SWISS-MODEL server and validated by PDBsum, ProSA-web, and ERRAT. For each protein, the percentage of amino acids in allowed regions was calculated by summing the values corresponding to “most favored”, “additional allowed”, and “generously allowed” regions obtained via PDBsum. The results showed that the structures of p53 and Casp9 reached 100% in allowed regions, while Casp3 reached 98.7%.

According to ProSA-web analysis, all three modeled proteins exhibited negative scores, ranging from −6.61 to −7.45, indicative of high-quality structural models. Additionally, the Z-score analysis showed that the values obtained for the modeled proteins matched those of crystallized proteins, further supporting the validity of the models. Based on the global energy of the proteins, all amino acids, except four in the case of Casp3, displayed values below zero, suggesting high structural quality. Finally, ERRAT analysis showed scores ≥ 70% for the modeled proteins (Supplementary Figure S2 and Table 7). Together, these results confirm the excellent structural quality of the modeled proteins, ensuring their reliability for molecular docking studies.

### 3.6. Prediction of Pockets

Our results showed that the analyzed proteins have multiple pockets in their 3D structures. However, we selected the first pocket in each case, as it exhibited the highest probability scores—all above 75%. The analysis showed that the pocket of p53 consists of 26 amino acids, the pocket of Casp3 contains 18 amino acids, and the pocket of Casp9 is composed of 19 amino acids, as detailed in Table S1.

### 3.7. BFNB Metabolites as Potential Modulators of p53, Casp3, and Casp9 Activity

**Figure 6:**
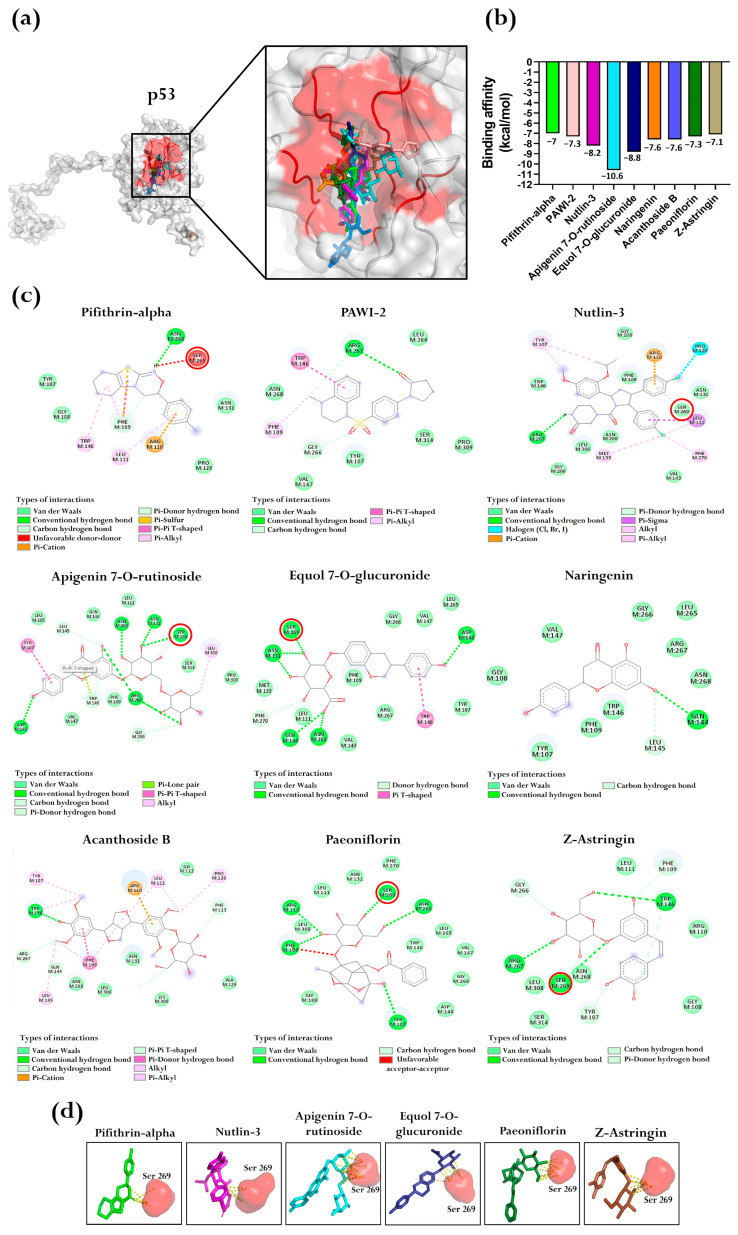
Molecular docking between inhibitors and BFNB metabolites with the p53 protein. (**a**) Representation of ligands bound to the p53 pocket, showing a close-up of the key interactions. The amino acids constituting the predicted pocket are indicated in red. (**b**) Comparative graph of binding affinities of the inhibitors and the BFNB metabolites with the highest affinity. In all cases, conformer 1 was selected for the comparative analysis. The bar colors in the graph correspond to the colors of the ligands represented in (**a**). (**c**) Two-dimensional diagrams of intermolecular interactions between the protein and the ligands, highlighting Ser 269 (indicated with red circles) as a key residue in ligand binding. (**d**) Visualization of polar interactions (yellow dashed lines) between the ligands and the Ser 269 residue, emphasizing its importance in stabilizing the protein–ligand complex.

Through blind molecular docking, we identified that reference inhibitors pifithrin-alpha, PAWI-2, and nutlin-3 bound to the predicted pocket of p53 with binding affinities of −7.0, −7.3, and −8.2 kcal/mol, respectively. Interestingly, six BFNB metabolites demonstrated comparable or more favorable (more negative) binding affinities relative to those of the reference inhibitors. These metabolites included apigenin 7-O-rutinoside, equol 7-O-glucuronide, naringenin, acanthoside B, paeoniflorin, and Z-astringin. Among them, apigenin 7-O-rutinoside and equol 7-O-glucuronide stood out, with binding affinities of −10.6 and −8.8, respectively, surpassing those of the reference inhibitors (Figure 6a,b).

Analysis of intermolecular interactions for the metabolites bound to the p53 pocket identified multiple interaction types, including van der Waals interactions, conventional hydrogen bonds, carbon–hydrogen bonds, Pi–cation interactions, Pi–hydrogen donor bonds, Pi–sulfur interactions, Pi–Pi stacking, T-shaped Pi–Pi stacking, Pi–alkyl interactions, Pi–sigma interactions, alkyl bonds, and Pi–lone pair bonds (Figure 6c).

Bauer et al. [46], previously identified Ser 220 of p53 as a critical residue for ligand interactions, contributing significantly to the functional modulation of the protein. In our analysis, given that our modeled structure corresponds to a complete protein, we confirmed, via sequence alignment, that this residue corresponds to amino acid 269. Interestingly, the intermolecular analysis conducted with Discovery Studio (Figure 6c) showed specific interactions between Ser 269 and the ligands. Additionally, PyMol analysis (Figure 6d) revealed multiple interactomic contacts between the ligands and Ser 269, except for the inhibitor PAWI-2, naringenin, and acanthoside B.

**Figure 7:**
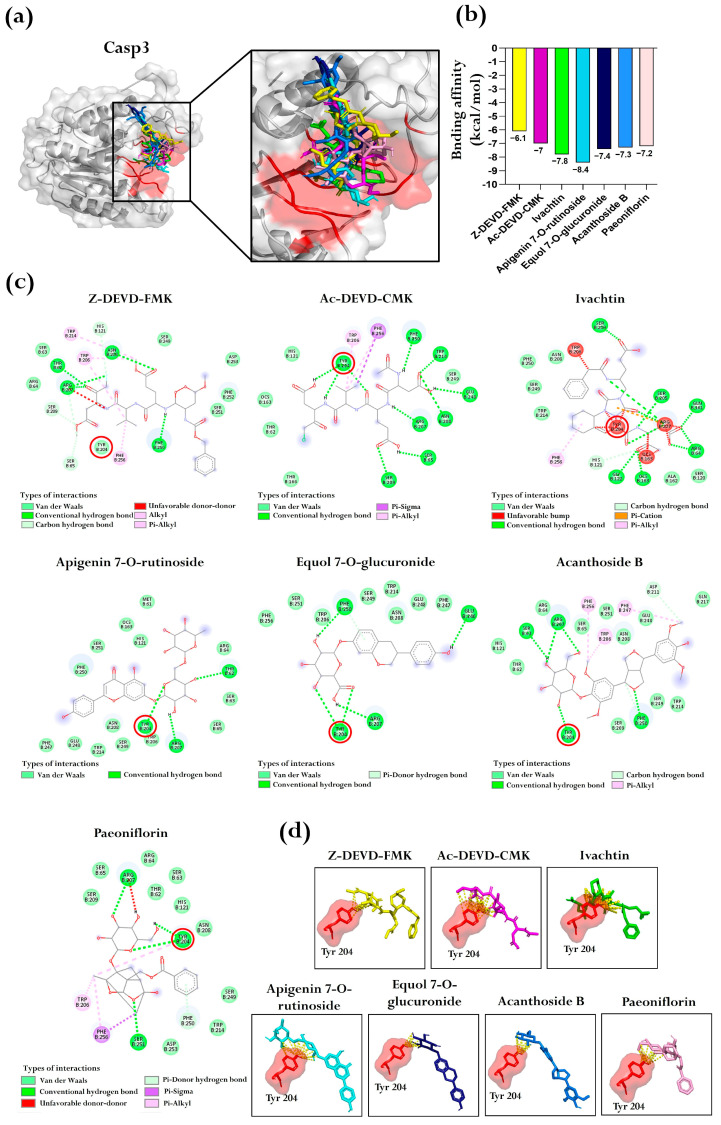
Molecular docking between inhibitors and BFNB metabolites with Casp3. (**a**) Representation of ligands bound to the predicted pocket of Casp3, including a close-up view highlighting ligand interactions. The amino acids constituting the predicted pocket are shown in red. (**b**) Comparative graph of binding affinities of the inhibitors and the BFNB metabolites with the highest affinity. In all cases, conformer 1 was selected for the comparative analysis. The bar colors in the graph correspond to the colors of the ligands represented in (**a**). (**c**) Two-dimensional diagrams of intermolecular protein–ligand interactions, highlighting Tyr 204 (indicated with red circles) as a key residue for ligand binding. (**d**) Visualization of specific ligand interactions with the Tyr 204 residue (yellow dashed lines), emphasizing its importance in stabilizing the protein–ligand complex.

Similarly, blind molecular docking studies were performed for Casp3. Three inhibitors (Z-DEVD-FMK, Ac-DEVD-CMK, and Ivachtin) were found to bind to the predicted pocket, with binding affinity ranging from −6.1 to −7.8 kcal/mol. Within this range, four BFNB metabolites were identified with comparable or better binding affinity, including apigenin 7-O-rutinoside, equol 7-O-glucuronide, acanthoside B, and paeoniflorin. Notably, apigenin 7-O-rutinoside exhibited the best binding affinity, with a value of −8.4 kcal/mol (Figure 7a,b).

The intermolecular analysis demonstrated that both the inhibitors and BFNB metabolites engage through various types of bonds, similar to those observed for p53, including van der Waals forces, conventional hydrogen bonds, and Pi interactions, among others (Figure 7c). Furthermore, Ganesan et al. [47], reported that Tyr 204 plays a crucial role in interactions modulating Casp3 functionality. Upon evaluating this residue, we found that all three inhibitors and the four BFNB metabolites formed multiple intermolecular interactions with Tyr 204, reinforcing its importance in stabilizing the formed complexes (Figure 7d).

**Figure 8:**
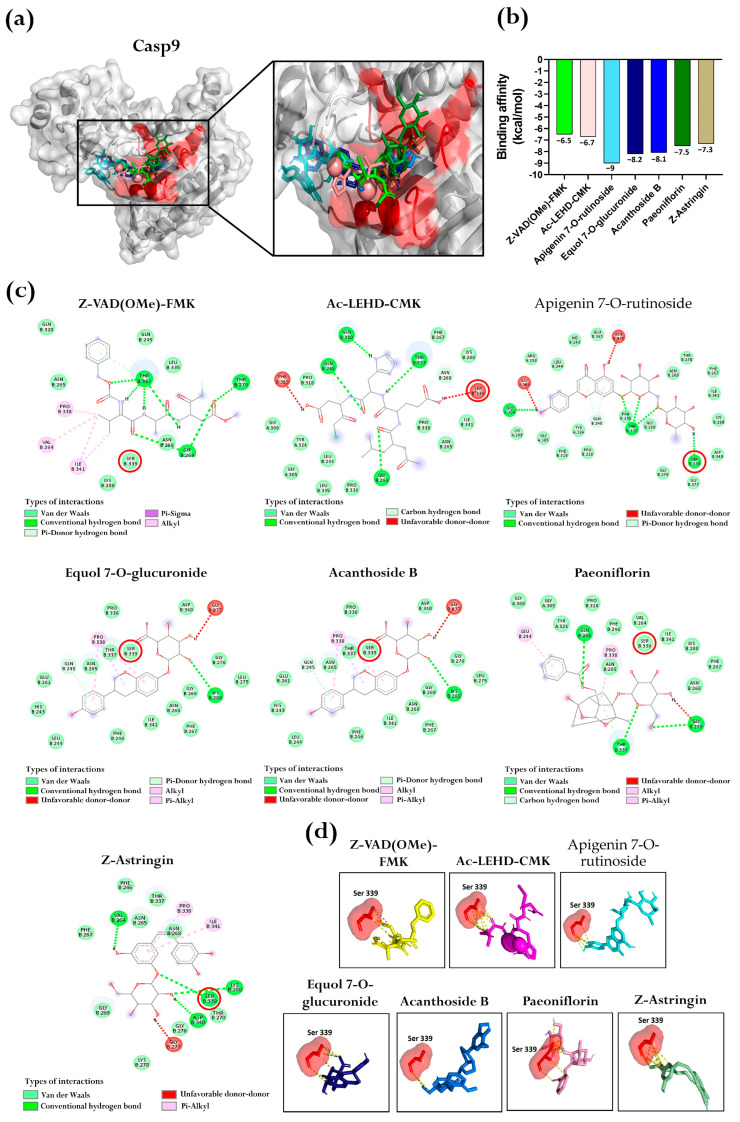
Molecular docking between inhibitors and BFNB metabolites with Casp9. (**a**) Representation of ligands bound to the predicted pocket of Casp9, including a close-up view highlighting ligand interactions. The amino acids constituting the predicted pocket are shown in red. (**b**) Comparative graph of binding affinities of the inhibitors and the BFNB metabolites with the highest affinity. In all cases, conformer 1 was selected for the comparative analysis. The bar colors in the graph correspond to the colors of the ligands represented in (**a**). (**c**) Two-dimensional diagrams of intermolecular protein–ligand interactions, highlighting Ser 339 (indicated with red circles) as a key residue for ligand binding. (**d**) Visualization of specific ligand interactions with the Ser 339 residue (yellow dashed lines), emphasizing its importance in stabilizing the protein–ligand complex.

For Casp9, the reference inhibitors, Z-VAD(OMe)-FMK and Ac-LEHD-CMK, were found to bind to the protein pocket with binding affinities of −6.5 and −6.7 kcal/mol, respectively. Interestingly, five BFNB metabolites exhibited more favorable binding affinity, ranging from −7.3 to −9 kcal/mol. Among them, apigenin 7-O-rutinoside showed the best binding affinity (−9 kcal/mol), followed by equol 7-O-glucuronide (−8.2 kcal/mol) (Figure 8a,b).

The intermolecular interaction analysis identified various interaction types similar to those observed for p53 and Casp3 (Figure 8c). Dall et al. [48] reported that Ser 215 plays a key role in ligand binding to the Casp9 pocket. Consistently, our results showed that Ser 339 is involved in multiple interactions with the utilized inhibitors and BFNB metabolites (Figure 8d).

As a complementary approach, a third protein–ligand interaction analysis was conducted using the PLIP server to enrich the information on the interactions. This analysis showed that, in addition to the previously identified interactions, the ligands bound to p53 (Supplementary Table S2), Casp3 (Supplementary Table S3), and Casp9 (Supplementary Table S4) also exhibited hydrophobic interactions and, in some cases, interactions through salt bridges. Consistently, the key amino acids in each protein were identified as critical interaction points.

### 3.8. Interactome Analysis and Enrichment of Apoptosis-Related Molecular Pathways

**Figure 9:**
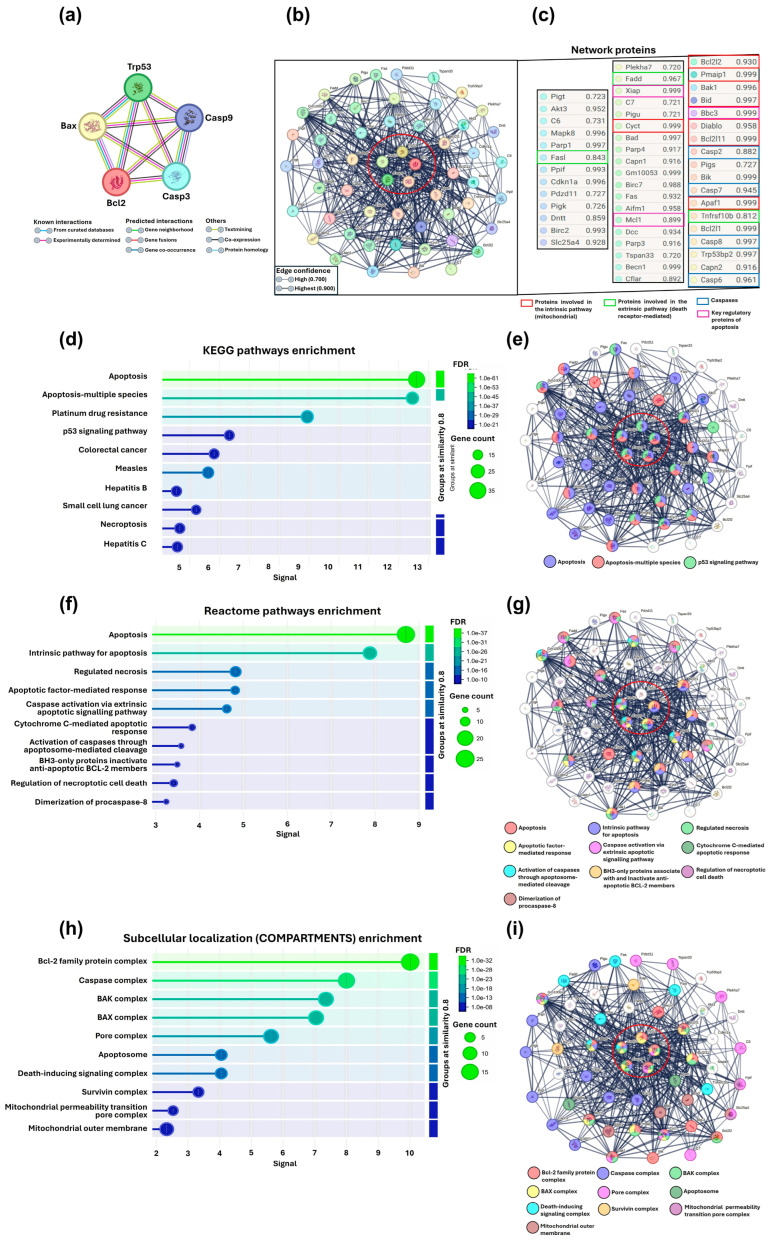
Interactome analysis and pathway enrichment of apoptosis-related proteins. (**a**) Initial interactome generated for the proteins evaluated in vivo, including p53, Casp3, Casp9, Bax, and Bcl-2. (**b**,**c**) Expanded interactome generated by comparing the five proteins of interest with the M. musculus proteome. The red circle encompasses the five proteins of interest, while the numbers represent interaction probabilities ≥70% and the colored squares show the key proteins involved in the regulation of apoptosis. (**d**) KEGG molecular pathway enrichment and (**e**) its associated interactome. (**f**) Reactome pathway enrichment and (**g**) its related interactome. (**h**) COMPARTMENTS pathway enrichment and (**i**) its corresponding interactome. In the interactomes generated during enrichment analyses, colored circles indicate key proteins from apoptotic pathways within the main clusters, emphasizing their functional relationships. The False Discovery Rate (FDR) is represented by a color scale (greener shades indicate higher significance), and circle size reflects the number of genes associated with each pathway. A stronger signal along the *X*-axis indicates greater enrichment, signifying a higher representation of associated genes compared to random expectations.

The interactomic analysis illustrated that the proteins studied in vivo via Western blot (p53, Casp3, Casp9, Bax, and Bcl-2) exhibited a close association within the generated network (Figure 9a), highlighting their collective involvement in apoptosis-related processes.

Expanding the interactomic network with the *M. musculus* proteome revealed 50 additional proteins with interaction probabilities ≥ 70%, resulting in a network comprising 55 nodes, 429 interactions, an average degree of 15.6 connections per protein, and a local clustering coefficient of 0.792. These parameters indicate that the network is densely connected and functionally coherent, highlighting potential key regulators of apoptosis.

Among the identified proteins, several were involved in the intrinsic (mitochondrial) pathway, such as Apaf1, Bak1, Bid, Diablo, Cyct, Bcl-2l2, and Bcl-2l1, as well as in the extrinsic (death receptor-mediated) pathway, including Fadd, Tnfrsf10b, and Fasl. Additionally, multiple caspases were identified, including Casp2, Casp6, Casp7, and Casp8. Key regulators of apoptosis, such as Bbc3, Mcl1, and Xiap, were also identified. These proteins represent critical checkpoints that determine whether a cell commits to apoptosis or survives under different stimuli (Figure 9b,c).

These proteins are involved in regulating the balance between cell survival and apoptosis, thereby strengthening our interactomic analysis across both platforms.

To complement the interactome analysis, we performed pathway enrichment to associate the generated network with the KEGG, Reactome, and COMPARTMENTS databases, considering only the top 10 pathways in each enrichment analysis.

In KEGG, we identified critical pathways involved in apoptosis, p53 signaling, and cell cycle regulation, which are fundamental for controlling growth, cell survival, and DNA damage response. The largest group of genes of interest corresponded to apoptosis and p53 signaling pathways (Figure 9d,e).

In the Reactome database, the most prominent gene groups were associated with apoptosis, including the intrinsic apoptosis pathway, regulated necrosis, apoptotic factor-mediated responses, and caspase activation through extrinsic signaling (Figure 9f,g).

Finally, in COMPARTMENTS, critical complexes and structures involved in apoptosis regulation were identified, such as the balance between pro- and anti-apoptotic signals, caspase activation, mitochondrial permeabilization, and apoptosome formation. These results underscore the importance of mitochondria and membranes as key sites in the regulation of apoptosis. The largest gene groups included Bcl-2 family complexes, caspase complexes, BAK and Bax complexes, the pore complex, the apoptosome, and death-inducing signaling complexes (Figure 9h,i).

As a complementary approach, we performed an interactomic and functional enrichment analysis with the GeneMANIA server, which is based on gene-level associations, in contrast to STRING, which focuses on protein-level interactions. This analysis highlighted a clear association among the five genes under evaluation. Upon expanding the network and performing functional enrichment, we observed further alignment with genes involved in the intrinsic and extrinsic apoptotic pathways, as well as regulators of cytochrome C release. Additionally, we identified seven genes not detected in STRING: *Nras*, *Hspd1*, *Mdm2*, *Bnip3*, *Dusp26*, *Pbk*, and *Moap1* (Supplementary Figure S3). These genes are involved in regulating the balance between cell survival and apoptosis, thereby strengthening our interactomic analysis across both platforms.

## 4. Discussion

In our study, through in silico predictions, we identified metabolites in the BFNB formulation associated with alopecia, hair fiber fragility, and apoptosis-related processes, including the modulation of intrinsic and extrinsic pathways through key proteins such as p53, Casp3, and Casp8, highlighting their multifaceted therapeutic potential.

To evaluate whether BFNB possesses the apoptosis-modulating effect predicted in silico, C57BL/6 mice were treated topically with BFNB, and samples were collected 15 days post treatment to analyze the expression of key proteins involved in the intrinsic apoptosis pathway, including p53, Casp3-p11, Casp9-p10, Bax, and Bcl-2. This selection was based on extensive evidence highlighting the p53-mediated pathway as a crucial regulator of the phases of the hair growth cycle [16,49].

Notably, protein p53, known as the “guardian of the genome”, plays a central role in regulating apoptosis, cell cycle control, and the cellular stress response [50]. In the context of hair growth, p53 has a dual function: while its activation promotes apoptosis in hair follicle cells during the catagen phase, it also contributes to the maintenance of the balance required for follicular renewal [51]. Overexpression or dysfunction of p53 can disrupt this balance, leading to fragility or hair loss [16,52].

Interestingly, our results show that p53 expression is significantly reduced in skin samples from the dorsal region and head of mice treated with BNFB compared to the control group (vehicle), as evidenced by Western blot assays. These findings are consistent with previous studies, such as those by Chen et al. [53], which demonstrated that topical Tetrahydroxystilbene Glucoside (TSG) significantly decreased p53 expression in mice following depilation, promoting follicular regeneration. Similarly, p53 inhibition with pifithrin-α also favored hair regeneration, albeit less effectively than TSG. Additionally, reduced p53 expression was observed in the same murine model used in our study and in samples from alopecic humans.

Additionally, El-Domyati et al. [54] demonstrated that in humans with androgenetic alopecia (AGA), elevated p53 levels in balding frontal areas correlate with reduced PCNA expression, linking the apoptosis–proliferation axis to hair loss. In contrast, mice treated with our BFNB formulation showed reduced p53 levels and increased PCNA expression [27], highlighting its potential to modulate this axis and promote hair regrowth.

In another study, Botchkarev et al. [51] demonstrated the critical role of p53 in regulating the hair cycle. They showed that mice with a *p53* gene deletion experienced a significant delay in hair follicle regression during the catagen phase, accompanied by reduced apoptosis in hair follicle matrix cells and increased expression of anti-apoptotic protein Bcl-2. Furthermore, a decrease in the expression of Bax and IGF-BP3, both regulated by p53 and essential for apoptosis, was observed. These findings demonstrated that p53 acts as a modulator of apoptosis during physiological hair follicle regression, underscoring its role in determining the duration of the catagen phase and controlling the hair cycle.

In our study, Western blot analysis demonstrated that fifteen days of BFNB treatment significantly reduced the protein expression levels of Casp3-p11, Casp9-p10, and Bax. In contrast, a notable increase in Bcl-2 expression was observed. These results suggest that BFNB modulates the balance between pro-apoptotic and anti-apoptotic signals, favoring cell survival in treated areas and supporting its potential as a hair regrowth therapy.

These results are in agreement with those of several other studies, including the report by Chen et al. [53], which demonstrated that, in the same murine model used in our study, two weeks of TSG treatment significantly reduced the levels of activated pro-apoptotic caspases (Casp3 and Casp9) and Bax expression while increasing Bcl-2 levels.

Similarly, Wang et al. [55] reported that policosanol, a compound derived from Chinese wax, exhibited anti-apoptotic effects in a Kunming mouse model of androgenetic alopecia induced by testosterone. In their study, policosanol reduced pro-apoptotic markers such as TGF-β2, Casp9, Casp3, and Bax while increasing Bcl-2 expression.

Likewise, Heon-Sub et al. [56] documented the apoptosis-inhibitory effects of ginsenoside F2, a ginseng-derived compound. This compound promoted the proliferation of human hair follicle dermal papilla cells (HFDPCs) and keratinocytes (HaCaT), suppressed hair cell apoptosis, and prevented premature catagen-phase entry in a C57BL/6 mouse model of dihydrotestosterone-induced alopecia. Treatment with ginsenoside F2 reduced pro-apoptotic markers, including TGF-β2, activated Casp3, Bax, and Casp12, while increasing Bcl-2 expression.

Similarly, Kim et al. [57] demonstrated that formononetin, a compound derived from red clover, promoted the regeneration of miniaturized hair follicles in C57BL/6 mice. After fourteen days of topical application, formononetin restored hair follicles to their normal size and stimulated the growth of new hair shafts. This effect was attributed to the inhibition of the Fas/FasL pathway, which led to decreased activation of pro-apoptotic caspases (Casp8 and Casp3), Bax, and p53, along with increased Bcl-2 expression.

The set of these reports suggests that BFNB has a similar mechanism for regulating apoptotic balance, favoring cell survival and promoting hair growth in C57BL mice.

The inhibition of apoptosis in key cells, such as follicular matrix keratinocytes and dermal papilla cells, has proven to be an effective mechanism for prolonging the anagen phase, during which active hair growth occurs. This prolongation not only strengthens the hair cycle but also prevents premature entry into the catagen phase, promoting more robust and sustained hair growth [16].

In our investigation, the immunohistochemistry results showed that both BFNB and minoxidil significantly reduced the expression of apoptosis biomarkers (p53, Casp3-p11, and Casp9-p10) in dermal cells, the follicular bulb, and surrounding follicular regions. This reduction suggests lower apoptotic activity, consistent with histological observations showing more hair follicles with increased thickness and length, which are characteristic of the anagen phase of the hair cycle. These findings indicate that these treatments modulate apoptosis pathways, potentially prolonging the anagen phase and promoting growth.

Additionally, our results are consistent with those reported by Chen et al. [53], who demonstrated, via TUNEL assays, that TSG effectively inhibits apoptosis in dermal and follicular bulb cells, showing greater anti-apoptotic activity than pifithrin-α and minoxidil. In contrast, Luanpitpon et al. [58] reported that cisplatin induces ROS, activates caspases, and triggers apoptosis in dermal papilla cells and keratinocytes, promoting the catagen phase and reducing hair growth. Notably, inhibiting apoptosis with antioxidants was shown to prevent this effect, promoting the anagen phase and enhancing hair growth.

Another study highlighting the role of apoptosis in hair growth was conducted by Miao et al. [59], who evaluated the effects of 6-gingerol, an active compound in ginger (*Zingiber officinale*). They found that it inhibits hair growth by inducing apoptosis in dermal and dermal papilla cells, as evidenced by decreased Bcl-2 expression and increased Bax levels. Its topical application in C57BL/6 mice reduced hair follicle density and prolonged the telogen phase, underscoring its impact on hair cycle modulation.

Our results in the murine model are promising and provide a solid foundation for extrapolation to humans, as caspases and key proteins like Bcl-2 and Bax are known to be dysregulated in individuals with alopecia, affecting apoptosis and follicular survival processes and consequently impairing hair growth [16].

To bridge the in silico and in vivo results and determine which BNFB metabolites might modulate p53, Casp3, and Casp9, we performed 3D structural modeling of these proteins and blind docking simulations with AutoDock Vina. By comparing the BNFB metabolites with reference inhibitory compounds, we identified specific interactions and binding affinities that support the observations from the in vivo assays.

In our analysis, blind docking simulations showed that, out of the sixteen metabolites present in the BFNB bioactive fraction, only six have the potential to modulate key proteins related to apoptosis. Interestingly, naringenin, the most abundant metabolite in the bioactive fraction (29.2%), binds exclusively to p53, suggesting a specific role in the modulation of this key target in the intrinsic apoptotic pathway. Equol 7-O-glucuronide, the second most abundant metabolite (19.3%), along with paeoniflorin (8.2%, third place) and apigenin 7-O-rutinoside (5.6%, fifth place), showed high potential by interacting with the predicted pockets of p53, Casp3, and Casp9. In contrast, Z-astringin, one of the least abundant metabolites (2.4%, eleventh place), binds specifically to Casp9. All these metabolites exhibited binding affinities comparable to or better than the reference inhibitors, highlighting their unique roles in the modulation of the apoptotic pathway and, consequently, in the regulation of hair growth. Notably, some metabolites showed strong affinities for p53, Casp3, and Casp9, suggesting a synergistic effect within the intrinsic apoptosis pathway. These results are consistent with predictions of the molecular activities of these metabolites, highlighting their potential to inhibit apoptosis and promote follicular health.

Although there are no specific reports on the involvement of these metabolites in hair growth, various studies have documented their ability to modulate apoptosis in different contexts, both in vitro and in vivo, using cell lines and murine models unrelated to hair growth [60,61,62,63]. A significant exception is naringenin, which has been shown to promote hair growth by regulating proliferation and apoptosis pathways in dermal papilla cells, keratinocytes, and C57BL/6 mice [64,65]. Additionally, this metabolite has been reported to enhance hair growth, either as a standalone treatment or in combination with minoxidil [66]. Given that naringenin is the predominant metabolite in the bioactive fraction of *B. procumbens* [28] present in BNFB, we believe it may play a key role in promoting hair growth, acting independently or synergistically with other metabolites in the formulation.

In our study, we integrated interactomic and functional enrichment analyses to map protein interactions and identify key molecules involved in apoptosis and hair growth, consistent with previous reports [67,68]. These analyses revealed significant associations between the proteins evaluated in vivo (p53, Casp3, Casp9, Bax, and Bcl-2) and critical regulators of apoptosis. Specifically, within the intrinsic (mitochondrial) pathway, proteins such as Apaf1, which activates Casp9 through apoptosome formation [69]; Bak1 and Bid, which promote cytochrome C release [70]; Diablo, which antagonizes caspase inhibitors [70]; and cytochrome C, which activates Apaf1 upon mitochondrial release [71], were highlighted. Furthermore, anti-apoptotic regulators such as Bcl-2l2 and Bcl-2l1, which inhibit the actions of pro-apoptotic proteins like Bax and Bak [72], underscored their critical role in maintaining the balance between pro- and anti-apoptotic signals.

In the extrinsic pathway (mediated by death receptors), key proteins were identified, including Fadd, which recruits Casp8 to the DISC complex [73]; Tnfrsf10b (TRAIL receptor 2), which mediates apoptotic signals induced by TRAIL [74]; and Fasl, which activates the Fas receptor to initiate the apoptotic cascade [75]. Among the caspases, Casp8, an initiator in the extrinsic pathway [76]; Casp9, activated by the apoptosome in the intrinsic pathway [77]; and Casp3 and Casp7, executors in the final stage of apoptosis [78], were identified. Additionally, Casp2 was found to be involved in the genotoxic damage response and PIDDosome formation [79], while Casp6 participates in the degradation of structural proteins [80]. Regulatory proteins were also found, including Bbc3 (PUMA), which mediates p53-dependent apoptosis [81]; Mcl1, which modulates cell survival through interactions with Bcl-2 family members [82]; and Xiap, which directly inhibits Casp3, Casp7, and Casp9 to modulate cellular sensitivity to apoptotic signals [83]. Functional enrichment analyses highlight the complexity of apoptosis regulation within the intrinsic and extrinsic pathways, providing a molecular basis suggesting how BFNB may influence apoptosis and promote hair growth through key regulators evaluated in vivo.

This study not only impacts hair regeneration but also offers insights into other processes where apoptosis plays a crucial role, including neurological [84], cardiac [85], renal [86], hepatic [87], autoimmune/inflammatory [88], oncological [89], and infectious diseases [90], among others.

Ongoing experiments aim to determine whether BFNB modulates the extrinsic apoptosis pathway by evaluating key proteins identified through in silico predictions. These studies also investigate the proliferation–apoptosis axis and its connection to processes such as the antioxidant system, lipid peroxidation, and angiogenesis, providing deeper insights into BFNB’s role in hair growth promotion. In parallel, we are optimizing the formulation and will assess its efficacy in models more representative of human biology, such as cultured hair follicles. Additionally, we will evaluate biomarkers from the molecular pathways identified in our studies to further substantiate its therapeutic potential.

**Figure 10:**
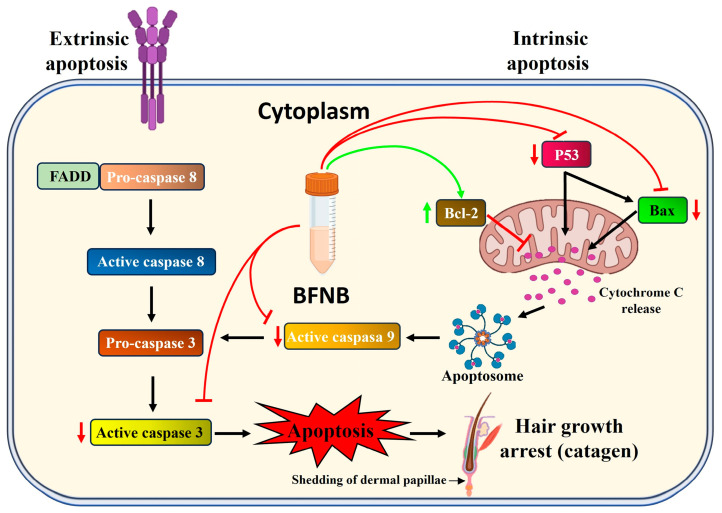
Proposed molecular mechanism modulated by BFNB in apoptosis regulation to promote hair growth. The results demonstrate that BFNB modulates the intrinsic apoptosis pathway by reducing the expression of p53, Casp3, Casp9, and Bax while increasing the expression of Bcl-2. These changes favor an anti-apoptotic balance in key dermal and hair follicle cells, promoting cell survival and hair growth. Furthermore, the predicted activities of BFNB metabolites and the interactomic and functional enrichment analyses suggest potential interactions not only with key proteins of the intrinsic pathway but also with extrinsic pathway proteins, such as Casp8, which would reinforce the comprehensive modulation of the apoptotic process by BFNB.

In Figure 10, we present a proposed molecular mechanism through which the BNFB formulation modulates apoptosis, specifically focusing on the intrinsic pathway. Our experimental findings suggest that BNFB regulates the apoptotic process in key cells within the dermis and follicular cells by reducing pro-apoptotic signals and increasing the expression of survival-associated factors. This molecular balance may be critical in creating a favorable environment that stimulates hair growth.

## 5. Conclusions

This study demonstrates the potential of BFNB, a nanostructured formulation of *Bacopa procumbens* with gold nanoparticles, to promote hair growth by regulating the intrinsic apoptosis pathway. This effect is achieved by reducing the expression of pro-apoptotic markers such as p53, Casp3, Casp9, and Bax while increasing the expression of anti-apoptotic markers like Bcl-2, thereby promoting cell survival within hair follicles. In silico analyses of the potential activities and molecular targets of BFNB metabolites, along with interactomic, functional enrichment, and molecular docking analyses, support the in vivo effects observed on the intrinsic apoptosis pathway. These findings highlight a coordinated regulatory mechanism involving multiple key nodes linked to the experimentally evaluated proteins, emphasizing their pivotal role in the modulation of apoptotic processes. This precise regulation of apoptosis-related molecular pathways underscores the potential of BFNB to stimulate hair growth, offering new perspectives for its therapeutic application in hair growth-related disorders.

## Figures and Tables

**Table 1:** Principal metabolites of the bioactive fraction of *B. procumbens* contained in the BFNB formulation.

No.	Metabolites	PubChem CID	SMILES
1	Naringenin	439246	C1[C@H](OC2=CC(=CC(=C2C1=O)O)O)C3=CC=C(C=C3)O
2	Equol 7-O-glucuronide	29979359	C1[C@H](COC2=C1C=CC(=C2)O[C@H]3[C@@H]([C@H]([C@@H]([C@H](O3)C(=O)O)O)O)O)C4=CC=C(C=C4)O
3	Peoniflorin	442534	C[C@]12C[C@@]3([C@@H]4C[C@]1([C@@]4([C@H](O2)O3)COC(=O)C5=CC=CC=C5)O[C@H]6[C@@H]([C@H]([C@@H]([C@H](O6)CO)O)O)O)O
4	m-Hydroxybenzoic acid	7420	C1=CC(=CC(=C1)O)C(=O)O
5	Apigenin 7-O-rutinoside	9851181	C[C@H]1[C@@H]([C@H]([C@H]([C@@H](O1)OC[C@@H]2[C@H]([C@@H]([C@H]([C@@H](O2)OC3=CC(=C4C(=C3)OC(=CC4=O)C5=CC=C(C=C5)O)O)O)O)O)O)O)O
6	Methyl ferulate	5357283	COC1=C(C=CC(=C1)/C=C/C(=O)OC)O
7	Acanthoside B	443024	COC1=CC(=CC(=C1O)OC)[C@@H]2[C@H]3CO[C@@H]([C@H]3CO2)C4=CC(=C(C(=C4)OC)O[C@H]5[C@@H]([C@H]([C@@H]([C@H](O5)CO)O)O)O)OC
8	o-Hydroxybenzoic acid	338	C1=CC=C(C(=C1)C(=O)O)O
9	Koparin	5318834	COC1=C(C(=C(C=C1)C2=COC3=C(C2=O)C=CC(=C3)O)O)O
10	Phloretic acid	10394	C1=CC(=CC=C1CCC(=O)O)O
11	Z-Astringin	16040016	C1=CC(=C(C=C1/C=C\C2=CC(=CC(=C2)O[C@H]3[C@@H]([C@H]([C@@H]([C@H](O3)CO)O)O)O)O)O)O
12	p-Hydroxybenzoic acid	135	C1=CC(=CC=C1C(=O)O)O
13	Stevenin	5321501	COC1=C(C=C2C(=CC(=O)OC2=C1)C3=CC(=CC=C3)O)O
14	Homovanillyl alcohol	16928	COC1=C(C=CC(=C1)CCO)O
15	Genistein	5280961	C1=CC(=CC=C1C2=COC3=CC(=CC(=C3C2=O)O)O)O
16	Catalposide	93039	C1=CO[C@H]([C@H]2[C@@H]1[C@@H]([C@H]3[C@@]2(O3)CO)OC(=O)C4=CC=C(C=C4)O)O[C@H]5[C@@H]([C@H]([C@@H]([C@H](O5)CO)O)O)O

**Table 2:** Reference inhibitors of proteins involved in apoptosis employed in our molecular docking studies.

Protein	Reference Inhibitor	PubChem CID	Reference
p53	Pifithrin-alpha	9929138	Sohn et al. [38]
	PAWI-2	89699521	Cheng et al. [39]
	Nutlin-3	216345	Dey et al. [40]
Casp3	Z-DEVD-FMK	73325061	Liu et al. [41]
	Ac-DEVD-CMK	9959259	Zakharova et al. [42]
	Ivachtin	11201705	Yosefzon et al. [43]
Casp9	Z-VAD(OMe)-FMK	5497174	Vu et al. [44]
	Ac-LEHD-CMK	16760347	Sodhi et al. [45]

**Table 3:** Fold change in expression levels of apoptosis-related proteins for BFNB compared to the control group and minoxidil, analyzed by Western blot.

	Dorsum		Head	
Protein	Control	Minoxidil	Control	Minoxidil
p53 (50 kDa)	−0.46	0.18	−0.42	0.21
p53 (53 kDa)	−0.50	0.28	−0.40	0.41
Casp3-p11	−0.45	0.10	−0.51	−0.22
Casp9-p10	−0.63	−0.24	−0.44	−0.34

The negative sign (−) indicates a decrease in expression.

**Table 4:** Fold change in expression levels of BFNB compared to the control group or minoxidil in survival/apoptosis axis markers, analyzed by Western blot.

	Dorsum		Head	
Protein	Control	Minoxidil	Control	Minoxidil
Bcl-2	4.23	2.43	2.77	1.20
Bax	−0.28	0.23	−0.39	−0.27
Bcl-2/Bax ratio	6.28	2.48	5.18	3.27

The negative sign (−) indicates a decrease in expression.

**Table 5:** Fold change in expression levels of proteins involved in apoptosis for BFNB compared to the control group or minoxidil, analyzed by immunohistochemistry in longitudinal sections.

Protein	Control	Minoxidil
p53	−0.68	−0.18
Casp3-p11	−0.61	−0.16
Casp9-p10	−0.66	−0.51

The negative sign (−) indicates a decrease in expression.

**Table 6:** Fold change in expression levels of proteins involved in apoptosis for BFNB compared to the control group or minoxidil, analyzed by immunohistochemistry in transverse sections.

Protein	Control	Minoxidil
p53	−0.48	0.22
Casp3-p11	−0.75	0.03
Casp9-p10	−0.79	−0.13

The negative sign (−) indicates a decrease in expression.

**Table 7:** Scores from the structural validation of modeled proteins utilizing different in silico tools.

SWISS-MODEL	PDBsum					ProSA-web	ERRAT
Protein	MFR	AAR	GAR	% of aa in PR	DR	Z-Score	OQF (%)
p53	70	26.4	3.6	100	0.0	−6.61	73.622
Casp3	85.3	12.0	1.3	98.7	1.3	−6.59	90.9091
Casp9	87.8	11.1	1.1	100	0.0	−7.45	94.7368

MFR: most favored regions; AAR: additional allowed regions; GAR: generously allowed regions; PR: permitted regions; DR: disallowed regions; OQF: overall quality factor; aa: amino acids.

## Data Availability

The datasets used and/or analyzed during the current study are available from the corresponding author upon reasonable request.

## Supplementary Materials

The following supporting information can be downloaded at: https://www.mdpi.com/article/10.3390/pharmaceutics17020222/s1, Figure S1. Major metabolites of the aqueous-ethanolic extract of *Bacopa procumbens*; Figure S2. 3D modeling and structural validation; Figure S3. Interactomic and functional enrichment analysis of genes involved in apoptosis; Table S1. Prediction of pockets in 3D proteins involved in apoptosis; Table S2. Analysis of the intermolecular interactions of inhibitors and BFNB metabolites at the binding pocket of p53; Table S3. Analysis of the intermolecular interactions of inhibitors and BFNB metabolites at the binding pocket of Casp3; Table S4. Analysis of the intermolecular interactions of inhibitors and BFNB metabolites at the binding pocket of Casp9.
