# Supplementary material for: Polyphenols from Bacopa procumbens Nanostructured with Gold Nanoparticles Stimulate Hair Growth Through Apoptosis Modulation in C57BL/6 Mice
Source: Pharmaceutics. 2025 Feb 9;17(2):222. doi: 10.3390/pharmaceutics17020222 (PMC11859437; doi:10.3390/pharmaceutics17020222)
Supplement: Supplementary file 1 [file pharmaceutics-17-00222-s001.zip › Supplementary Figure S1-S3.pdf]

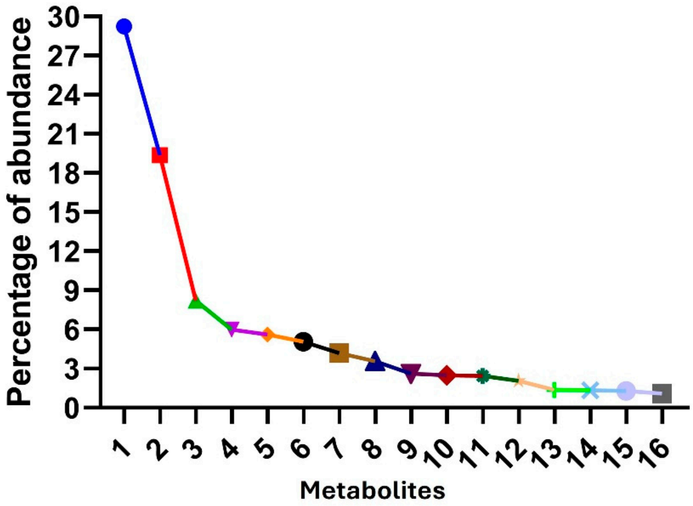

Supplementary Figure S1. Major metabolites of the aqueous-ethanolic extract of *Bacopa procumbens*. 1. Naringenin, 2. Equol 7-O-glucuronide, 3. Peoniflorin, 4. m-Hydroxybenzoic acid, 5. Apigenin 7-O-rutinoside, 6. Methyl ferulate, 7. Acanthoside B, 8. o-Hydroxybenzoic acid, 9. Koparin, 10. Phloretic acid, 11. Z-Astringin, 12. p-Hydroxybenzoic acid, 13. Stevenin, 14. Homovanillyl alcohol, 15. Genistein, 16. Catalposide.

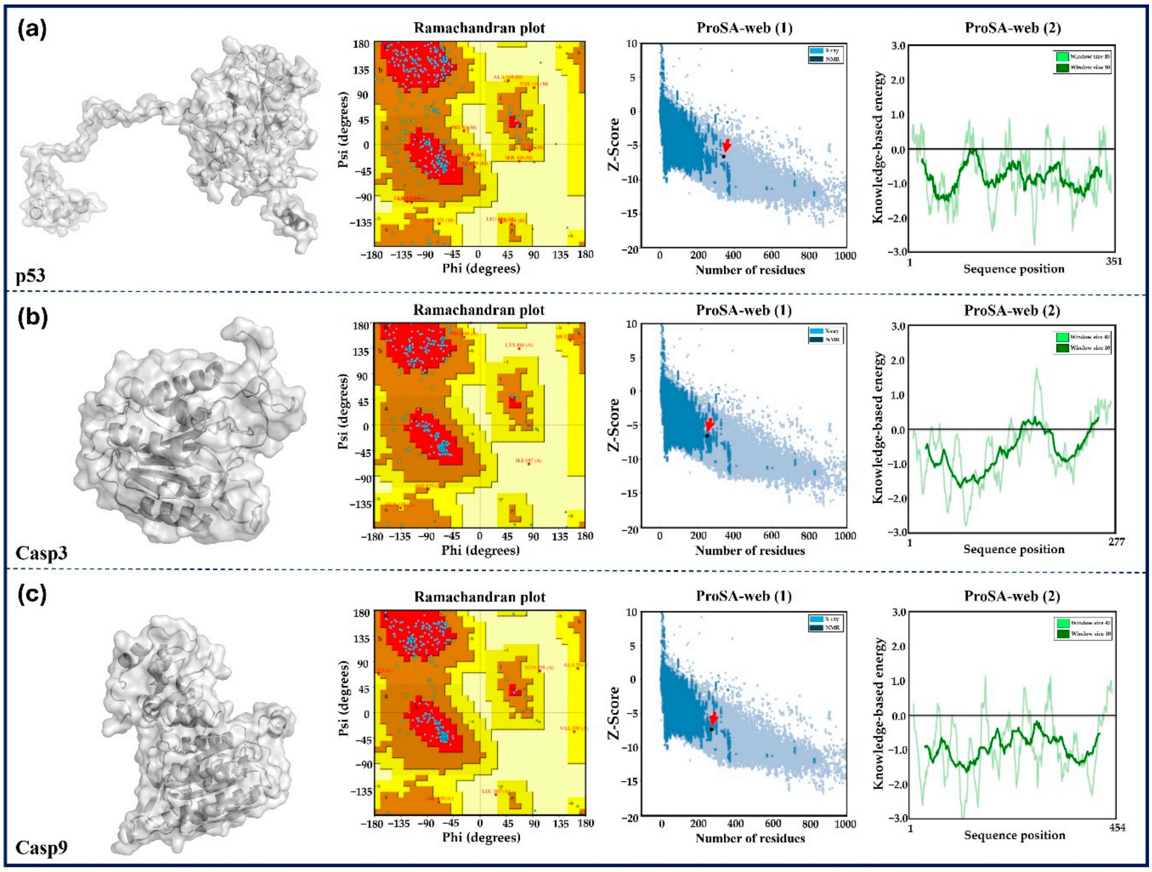

Supplementary Figure S2. 3D modeling and structural validation. The 3D modeling and structural validation of p53 (a), Casp3 (b), and Casp9 (c) are presented. For each protein, the figure includes the Ramachandran plot analysis and two graphs generated by ProSA-web: one showing the Z-score (ProSA-web 1) and another illustrating the global energy (ProSA-web 2). The red arrow indicates the Z-score obtained for each protein and its alignment with the Z-scores of crystallized proteins.

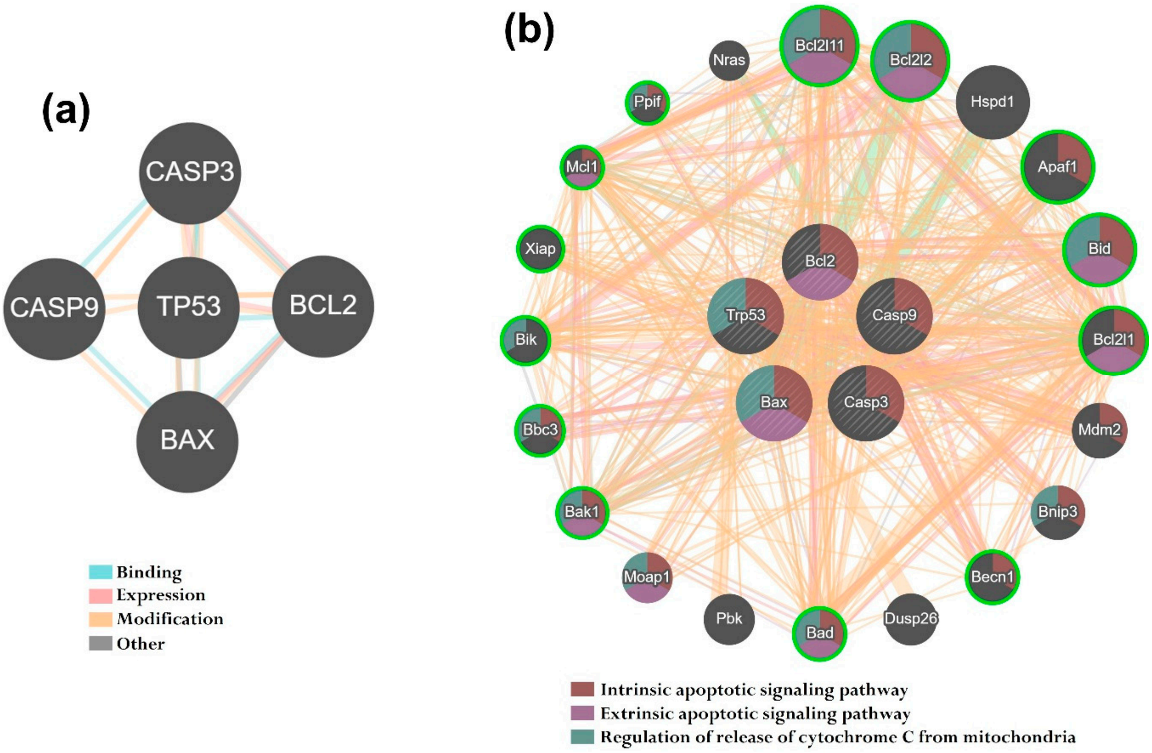

Supplementary Figure S3. Interactomic and functional enrichment analysis of genes involved in apoptosis. (a) Core network of five genes of interest generated using GeneMANIA. (b) Expanded network of the five genes of interest associated with the *M. musculus* proteome, including a functional enrichment analysis highlighting genes involved in the intrinsic and extrinsic apoptotic pathways, as well as the regulation of cytochrome C release. Genes highlighted with green borders indicate those that were also identified at the protein level in the interactomic analysis performed using STRING.
