# Supplementary material for: Polyphenols from Bacopa procumbens Nanostructured with Gold Nanoparticles Stimulate Hair Growth Through Apoptosis Modulation in C57BL/6 Mice
Source: Pharmaceutics. 2025 Feb 9;17(2):222. doi: 10.3390/pharmaceutics17020222 (PMC11859437; doi:10.3390/pharmaceutics17020222)
Supplement: Supplementary file 1 [file pharmaceutics-17-00222-s001.zip › Supplementary Table S1-S4.pdf]

Supplementary Table S1. Prediction of pockets in 3D proteins involved in apoptosis.

Proteins	Probability score (%)	No. of amino acids	Amino acids comprising the pocket
p53	83.4	26	Tyr (107), Gly (108), Phe (109), Arg (110), Gly (112), Asn (131), Thr (140), Cys (141), Pro (141), Val (142), Gln (143), Leu (144), Trp (145), Val (146), Asp (147), Ser (148), Leu (264), Leu (265), Gly (266), Arg (267), Asn (268), Ser (269), Leu (308), Pro (309), Ser (313), Ser (314)
Casp3	85	18	Met (61), Thr (62), Ser (63), Arg (64), Ser (65), Ser (120), His (121), Gly (122), Phe (128), Gln (161), Ala (162), Gly (165), Thr (166), Tyr (204), Ser (205), Trp (206), Arg (207), Phe (256)
Casp9	75.3	19	Tyr (153), Val (260), Glu (261), Ile (263), Val (264), Asn (265), Asn (268), Gly (269), Thr (270), Gly (276), Gly (277), Pro (279), Pro (336), Thr (337), Pro (338), Ser (339), Asp (340), Ile (341), Phe (42)

The numbers in parentheses represent the position of the amino acid within the 3D protein.

Supplementary Table S2. Analysis of the intermolecular interactions of inhibitors and BFNB metabolites at the binding pocket of p53.

		Type of interaction		
**Ligands**	**No. of total interactions**	**Hydrophobic**	**Hydrogen bonds**	**Salt bridges**
Pifithrin-alpha	4	Tyr (107), Phe (109), Leu (111), Trp (146)	-	-
PAWI-2	5	Tyr (107), Phe (109), Trp (146), Trp (146)	Arg (267)	-
Nutlin-3	3	Phe (109), Leu (111)	Arg (267)	-
Inhibitor chiral	5	Tyr (107), Phe (109), Phe (109), Asn (131), Trp (146)	-	-
Apigenin 7-rutinoside	14	Phe (109), Trp (146), Trp (146), Asp (148), Leu (308)	Asn (131), Gln (144), Trp (146), Asp (148), Arg (267), Arg (267), Arg (267), Ser (269), Ser (269)	-
Equol 7-O-glucuronide	8	Phe (109), Phe (109), Leu (111), Leu (111)	Asn (131), Asn (131), Gln (144), Asn (268)	-
Naringenin	7	Tyr (107), Phe (109), Phe (109), Trp (146)	Gln (144), Trp (146), Gly (266)	-
Acanthoside B	7	Tyr (107)	Arg (110), Pro (128), Trp (146), Trp (146)	Arg (110), Arg (110)
Koparin	3	Phe (109), Trp (146), Asp (148)	-	-
Paeoniflorin	14	Tyr (107), Phe (109), Trp (146), Trp (146), Asp (148), Leu (308)	Tyr (107), Phe (109), Arg (110), Leu (111), Asn (131), Trp (146), Ser (269), Ser (269)	-
Z-Astringin	8	Tyr (107), Phe (109), Phe (109)	Leu (111), Trp (146), Trp (146), Arg (267), Ser (269)	-

The occurrence of the same amino acid within the same intermolecular interaction group indicates its interaction with different regions of the ligand. The numbers in parentheses represent the position of the amino acid within the 3D protein structure, and the '-' symbol denotes the absence of that type of interaction.

Supplementary Table S3. Analysis of the intermolecular interactions of inhibitors and BFNB metabolites at the binding pocket of Casp3.

		Type of interaction		
**Ligands**	**No. of total interactions**	**Hydrophobic**	**Hydrogen bonds**	**Salt bridges**
Inhibitor I	14	Phe (250), Phe (250), Phe (256)	His (121), Gly (122), Gly (122), Glu (123), Gly (165), Gly (165), Ser (205), Thr (62), Phe (250), Phe (250)	Arg (207)
Inhibitor III	27	Trp (206)	His (121), Gly (122), Gly (122), Glu (123), Gly (165), Gly (165), Ser (205), Ser (65), Ser (65), His (121), Tyr (204), Tyr (204), Tyr (204), Tyr (204), Tyr (204), Arg (207), Arg (207), Arg (207), Arg (207), Asn (208), Ser (209), Trp (214), Glu (248), Phe (250), Phe (250)	Arg (207)
B92	27	Tyr (204), Tyr (204), Trp (206), Trp (206), Arg (207), Trp (214), Phe (256)	Arg (64), Arg (64), His (121), Gly (122), Gly (122), Gln (161), Tyr (204), Ser (205), Arg (207), Arg (207), Ser (209), His (121), Gly (122), Glu (123), Gly (165), Ser (205), Ser (205)	Arg (64), His (121), Arg (207)
Apigenin 7-rutinoside	17	Tyr (204), Trp (214)	His (121), Gly (122), Gly (122), Glu (123), Gly (165), Gly (165), Ser (205), Thr (62), Ser (63), His (121), Tyr (204), Arg (207)	His (121), Arg (207), Arg (207)
Equol 7-O-glucuronide	14	Trp (206), Phe (247), Phe (250)	His (121), Gly (122), Gly (122), Glu (123), Gly (165), Gly (165), Ser (205), Tyr (204), Asn (208), Glu (246), Phe (250)	-
Acanthoside B	16	Trp (206)	His (121), Gly (122), Gly (122), Glu (123), Gly (165), Gly (165), Ser (205), Thr (62), Ser (63), Ser (65), Ser (65), Tyr (204), Arg (207), Phe (250)	Arg (207)
Paeoniflorin	19	Trp (206), Trp (214), Phe (250), Phe (256), Phe (256)	His (121), Gly (122), Gly (122), Glu (123), Gly (165), Gly (165), Ser (205), Thr (62), Tyr (204), Arg (207), Arg (207), Phe (250), Ser (251)	Arg (207)

The occurrence of the same amino acid within the same intermolecular interaction group indicates its interaction with different regions of the ligand. The numbers in parentheses represent the position of the amino acid within the 3D protein structure, and the '-' symbol denotes the absence of that type of interaction.

Supplementary Table S4. Analysis of the intermolecular interactions of inhibitors and BFNB metabolites at the binding pocket of Casp9.

		Type of interaction		
**Ligands**	**No. of total interactions**	**Hydrophobic**	**Hydrogen bonds**	**Salt bridges**
ZVAD-FMK	7	Val (264), Thr (337)	Gly (269), Thr (270), Thr (337), Thr (337), Thr (337)	-
Inhibitor III	10	Pro (318), Leu (335), Pro (338)	Gln (245), Gly (269), Phe (319), Phe (319), Tyr (324), Thr (337), Ser (339)	-
Apigenin 7-rutinoside	10	Leu (244)	Gln (245), Asn (265), Asn (265), Phe (267), Gly (304), Gly (306), Thr (337), Ser (339), Asp (340)	-
Equol 7-O-glucuronide	10	Asn (268), Pro (338), Ile (341)	Gly (269), Gly (277), Lys (280), Thr (337), Ser (339), Ser (339)	Lys (280)
Acanthoside B	10	Leu (244)	Gln (245), Arg (258), Gly (269), Lys (280), Gly (304), Gly (306), Tyr (324), Thr (337), Thr (337)	-
Stevenin	11	Val (264), Asn (268), Pro (338), Ile (341), Ile (341)	Val (264), Phe (267), Gly (269), Gly (277), Lys (280), Asp (340)	-
Paeoniflorin	7	Leu (244), Leu (244), Tyr (324), Pro (338)	Gln (245), Gly (269), Thr (337)	-
Koparin	6	Pro (338), Ile (341)	Gly (277), Lys (280), Ser (339), Asp (340)	-
Z-Astringin	14	Asn (268), Pro (338), Pro (338)	Val (264), Phe (267), Asn (268), Gly (269), Thr (270), Gly (277), Lys (280), Ser (339), Asp (340), Asp (340)	Lys (280)

The occurrence of the same amino acid within the same intermolecular interaction group indicates its interaction with different regions of the ligand. The numbers in parentheses represent the position of the amino acid within the 3D protein structure, and the '-' symbol denotes the absence of that type of interaction.
